# FUE as the first surgical option for hair reconstruction on scalp and facial skin grafts – case report

**DOI:** 10.25122/jml-2023-0492

**Published:** 2024-02

**Authors:** Felix Mircea Popescu, Lidia Filip, Matei Popescu

**Affiliations:** 1 Department of Plastic and Reconstructive Surgery, Dr. Felix Hospital for Hair Reconstructive Surgery, Bucharest, Romania; 2 Department of Aesthetic Dermatology, M1 Med Beauty Clinic, Bucharest, Romania; 3 Research Department, Dr. Felix Hospital for Hair Reconstructive Surgery, Bucharest, Romania; 4 Nova Southeastern University, Florida, United States of America

**Keywords:** follicular unit excision, skin grafts, alopecia, post-combustion alopecia, hair reconstruction, eyebrow reconstruction, burns, FU, Follicular Unit, FUE, Follicular Unit Extraction

## Abstract

Post-combustion alopecia presents a complex medical challenge with implications spanning dermatological and psychiatric disorders. The use of hair transplantation has proven to be a significant improvement for this condition. However, the current management involves various techniques, each with advantages and disadvantages. Progressive skin expansions, surgical scar reduction, and skin grafts containing hair follicles yield unsatisfactory aesthetic outcomes and have limited applicability as a first-line treatment for fire victims. So far, follicular unit extraction (FUE) has proven to be one of the most versatile procedures in such cases, having the potential to restore a natural anatomical profile closely resembling the pre-traumatic appearance that led to the traumatic alopecia. Additionally, it contributes to the improvement of associated psychiatric comorbidities, facilitating proper social reintegration and enhancing overall quality of life. This report focuses on a case of post-combustion alopecia and severe facial distortion due to third-degree burns resulting in severe psychiatric comorbidities, which benefited from a proper social reintegration and improvement of the quality of life after three consecutive sessions of FUE for scalp and eyebrow hair.

## INTRODUCTION

Hair transplantation has become an increasingly standard practice in plastic surgery, and in recent years, the technique has improved significantly. Apart from purely aesthetic hair rejuvenation treatments, hair transplantation plays a special role in hair reconstruction for patients with scalp or facial trauma, aiding in social reintegration while improving the psychiatric comorbidities such as major depressive episodes and post-traumatic stress disorder, which frequently accompany any life-threatening event that leads to alopecia, such as severe burns [1]. In the context of limited surgical options that lead to satisfactory aesthetic outcomes, the follicular unit extraction (FUE) technique offers a personalized surgical experience for patients presenting with post-combustion alopecia and skin grafts [2].

## CASE PRESENTATION

**Figure 1:**
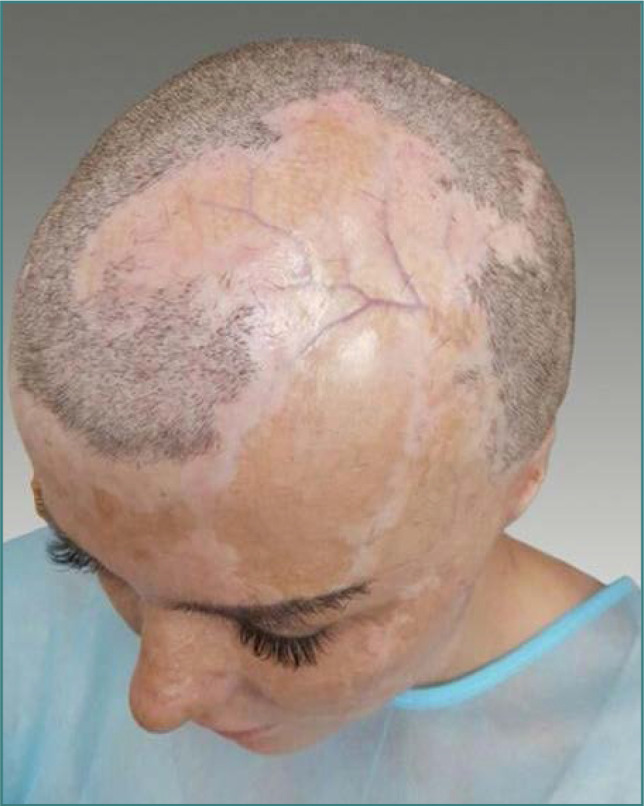
Healed frontal-parietal-lateral scalp skin grafts and cicatricial alopecia in a 27-year-old woman

A 27-year-old woman who survived third-degree burns covering 60% of her body after periods of induced coma and several resuscitations presented to our clinic with severe distortion of facial features and suicidal tendencies. The patient needed reconstruction with skin grafts not only to the face but also to the frontal-parietal-lateral scalp, with more than 100 cm^2^ of skin needing hair restoration (Figure 1).

We envisioned the FUE technique as the first choice for the following reasons: firstly, using the skin-reducing technique rendered difficulties, as the transplanted scalp area was large (110 x 140 mm). Secondly, micro-scars in the donor area, along with the presence of a large number of skin grafts, greatly limited the ability to extract the follicular unit (FU) via the FUE technique. Subsequently, progressive expansion and skin reduction were limited by the minimal tissue elasticity. Thirdly, multiple hypotrophic areas at the junction between healthy and grafted skin scalp showing fragility contraindicated the use of any other hair transplant technique besides FUE.

**Figure 2:**
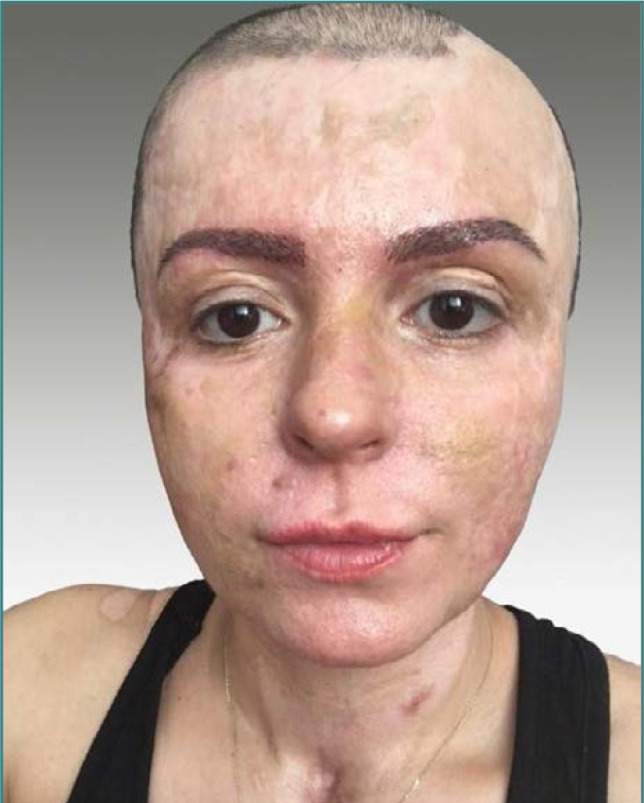
FUE eyebrow reconstruction – after two months

The whole hair reconstruction project was performed in 3 consecutive sessions spread apart at 6-month intervals, with one reconstructive session for eyebrows and two sessions for scalp restoration, using 6547 hair grafts (Figure 2). During the eyebrow restoration, we prioritized three key principles: achieving symmetry, replicating the original brow shape, and mimicking the natural hair growth direction. We chose an average density of 40-50 grafts per cm^2^ with an average ratio of 2.2 hairs per graft on the newly reconstructed surfaces. Areas that showed lower vascular perfusion, estimated by piercing the skin with a 19-gauge needle and observing the blood flow, were reconstructed with a lower density of 35-40 grafts per cm^2^ to ensure the viability of both the skin and hair grafts [3]. Extraction was made with a 0.8 mm diameter hand punch, and implantation was done with Choi pen implanters. Nevertheless, eyebrow restoration was not an easy process due to the presence of three superimposed different skin grafts, showing various degrees of tractional, slightly hypertrophic scarring.

The scalp project was also challenging due to seven prior skin graft attempts, with multiple rejections, causing concern and caution before starting FUE. Our objective was to define the face by recreating the frontotemporal hairline. The work surface included four different types of grafts, showing different degrees of hypotrophy and low perfusion at the skin-graft junction. Three months prior to transplantation, we increased the trophicity of the skin by using monthly micro-lipo-filling under poorly perfused areas, as well as adipocyte stem cells and growth factors, to accelerate vascular neogenesis.

**Figure 3:**
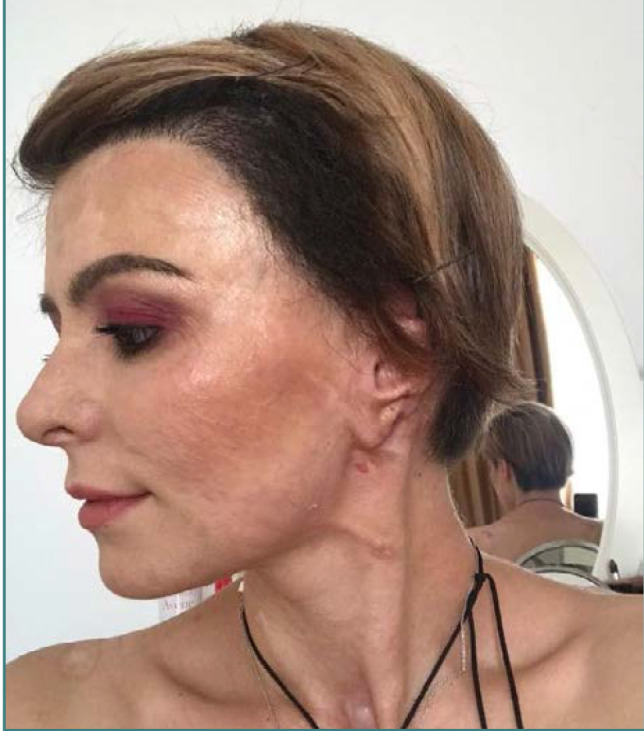
FUE scalp reconstruction – after two years

Extraction was done with 1.00 mm diameter manual punches, and implantation was realized with Choi pen implanters. Unfortunately, due to the fragile nature of the healing skin grafts and the limited vascular perfusion at the skin-graft junction, on day 15, an isolated 2/3 cm^2^ necrotic area developed on the frontoparietal scalp, instating the need for a preventive broad-spectrum antibiotic for seven days, followed by topical rifampicin for several weeks, until the forming granulation tissue allowed us to continue the FUE procedure. Months after successfully transplanting hair onto the skin grafts, we observed that the skin quality improved (Figure 3). The pathology report supports this by indicating good vascular perfusion of the FU and the perifollicular skin and a good sebaceous ratio per FU.

## DISCUSSION

Post-combustion alopecia is a problem with multiple medical implications, ranging from dermatological to psychiatric disorders, which can be significantly improved using hair transplantation. Currently, this is being managed through several techniques, each with advantages and disadvantages. Due to the fragile nature of the skin grafts and the fibrotic tissue developed in the burn wounds, choosing progressive skin expansions renders difficulties [4]. It is challenging to improve the expansion index, but reconstructing facial hair, such as eyebrows or beards, becomes impossible in these areas, similar to the scar reduction technique [5]. Using skin grafts containing hair follicles also shows unsatisfactory aesthetic outcomes due to the facial units’ mismatch in terms of hair and skin, quality and function [6]. As a result, these techniques show limited indications as first intention treatment for fire victims, thus making FUE the most versatile procedure in these cases. The FUE technique can restore a natural anatomical profile, very close to the pre-traumatic appearance that caused the cicatricial alopecia, while also improving the associated psychiatric comorbidities, leading to proper social reintegration. Prospects in the field of matrix cell culture will open up new horizons for improving not only the minimally invasive aspect of the procedure but also the quality of the grafts, thus having an impact on improving patients' quality of life.

## CONCLUSION

Hair transplantation has become widely accepted in plastic surgery, with notable advancements in recent years. Beyond its aesthetic benefits, hair transplantation plays a crucial part in the recovery process, contributing to social reintegration and addressing psychiatric comorbidities such as major depressive episodes and post-traumatic stress disorder. Our 27-year-old patient presented with severe depression and suicidal thoughts due to extensive burn trauma sustained after a significant life-threatening event. The face and the frontal-parietal-lateral scalp required important plastic surgery with skin grafts. The hair reconstruction of the scalp and the eyebrows required several sessions of an adapted form of the FUE technique, with varying hair graft density according to the degree of vascular perfusion of the skin grafts, as well as several sessions of prior micro-lipo-filling to increase the viability of the tissue. Several months following the successful transplantation of hair onto the skin grafts, we have noted an enhancement in the skin's quality, with microscopical indications of increased vascular perfusion of the FU and the perifollicular skin, along with a satisfactory sebaceous ratio per follicular unit.

## Data Availability

Further data are available from the corresponding author upon reasonable request.
